# α-proteobacteria synthesize biotin precursor pimeloyl-ACP using BioZ 3-ketoacyl-ACP synthase and lysine catabolism

**DOI:** 10.1038/s41467-020-19251-5

**Published:** 2020-11-05

**Authors:** Yuanyuan Hu, John E. Cronan

**Affiliations:** 1 Department of Biochemistry, University of Illinois at Urbana-Champaign, Urbana, IL USA; 2 Department of Microbiology, University of Illinois at Urbana-Champaign, Urbana, IL USA

**Keywords:** Enzyme mechanisms, Bacteria, Enzymes, Biosynthesis

## Abstract

Pimelic acid, a seven carbon α,ω-dicarboxylic acid (heptanedioic acid), is known to provide seven of the ten biotin carbon atoms including all those of the valeryl side chain. Distinct pimelate synthesis pathways were recently elucidated in *Escherichia coli* and *Bacillus subtilis* where fatty acid synthesis plus dedicated biotin enzymes produce the pimelate moiety. In contrast, the α-proteobacteria which include important plant and mammalian pathogens plus plant symbionts, lack all of the known pimelate synthesis genes and instead encode *bioZ* genes. Here we report a pathway in which BioZ proteins catalyze a 3-ketoacyl-acyl carrier protein (ACP) synthase III-like reaction to produce pimeloyl-ACP with five of the seven pimelate carbon atoms being derived from glutaryl-CoA, an intermediate in lysine degradation. *Agrobacterium tumefaciens* strains either deleted for *bioZ* or which encode a BioZ active site mutant are biotin auxotrophs, as are strains defective in CaiB which catalyzes glutaryl-CoA synthesis from glutarate and succinyl-CoA.

## Abstract (web summary)

Biosynthesis of biotin precursor, pimelate moiety, is elucidated in *Escherichia coli* and *Bacillus subtilis*, but not understood in alphaproteobacteria. Here, the authors show that BioZ, a 3-ketoacyl-ACP synthase, catalayses pimeloyl-thioester synthesis in alphaproteobacteria using malonyl-ACP and glutaryl-CoA that is derived from lysine degradation.

## Introduction

**Fig. 1:**
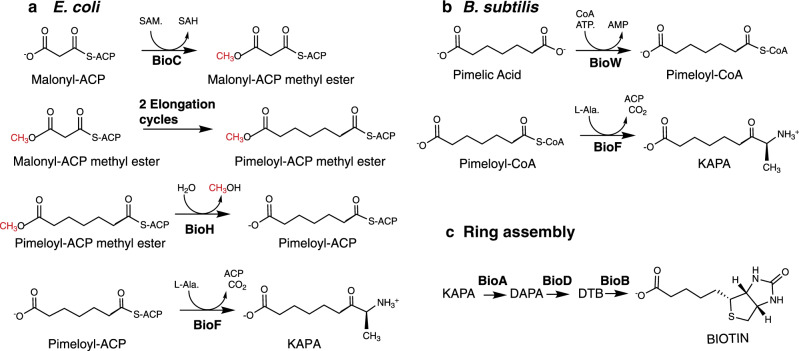
The known pathways of pimeloyl moiety synthesis. **a** In *E. coli* and (as inferred from genome analyses) many other bacteria, malonyl-ACP methyl ester replaces the usual acetyl primer of fatty-acid synthesis. After two cycles of chain elongation the methyl group is removed by BioH to give the BioF substrate, pimeloyl-ACP. (Red letters denote the methyl group donated by S-adenosyl-l-methionine (SAM) and subsequently released as methanol. **b** In *B. subtilis* the pimelate chain is also assembled by fatty-acid synthesis, but unlike *E. coli*, the pathway proceeds through a free pimelic acid intermediate that requires activation by BioW. **c** Schematic of the late steps in biotin synthesis. Systematic chemical nomenclature for KAPA is 7-keto-8-aminopelargomic acid whereas DAPA is 7,8-diaminononanoic acid. DTB is dethiobiotin.

Biotin (coenzyme R, vitamin H) is an essential cofactor for carboxylation, decarboxylation, and transcarboxylation reactions in several key metabolic pathways, such as gluconeogenesis, fatty acids synthesis, and branched chain amino-acid degradation^1,2^. Only bacteria, archea, fungi, and plants synthesize biotin. Biotin consists of a tetrahydroimidizalone ring fused with an organosulfur-containing tetrahydrothiophane ring that bears a valeric acid substituent, which originates from a pimelic acid (heptanedioic acid) moiety^3^. The enzymes that assemble the fused rings of biotin are conserved in biotin-producing bacteria, archea, plants, and fungi^4^ (Fig. 1). In contrast, diverse pathways exist for the synthesis of the pimelate moiety precursor. The known pathways, those of *Escherichia coli* and *Bacillus subtilis*, involve enzymes of fatty-acid synthesis^5,6^ (Fig. 1). In *E. coli* ^13^C-NMR analyses of the patterns of incorporation of various ^13^C-labeled biotin synthesis precursors demonstrated that the pimelic acid moiety is formed from acetate units incorporated in a head-to-tail manner^7,8^, the pattern seen in fatty acid and polyketide synthesis^9^. A similar head-to-tail pattern is seen in *B. subtilis* although the labeling pattern differs from that of *E. coli* because free pimelic acid is an intermediate^6^. In both cases the seventh pimelate carbon atom originates from CO_2_.

The most straightforward means to obtain a seven-carbon dicarboxylic acid is to oxidatively cleave a longer fatty acid. Indeed, the Gram-positive bacterium *B. subtilis* encodes a cytochrome P450 oxidoreductase called BioI that can cleave a long acyl-ACP chain into pimeloyl-ACP^10,11^. However, *B. subtilis* BioI is an enigma because it is not essential for biotin synthesis and makes a product that cannot be utilized by BioF, the next enzyme of the pathway^12^. Instead the pimeloyl-CoA ligase (BioW) plays a key role in biotin synthesis^12^ (Fig. 1b).

Another proposed approach to obtain the pimelate moiety is to condense three molecules of malonate in two decarboxylative Claisen-like condensations to obtain pimeloyl-ACP^13^. However, the hydrophobicity of the active sites evident in crystal structures of fatty-acid synthetic enzymes argued that the postulated free carboxyl group would not be tolerated. This dilemma is avoided in many bacteria by the pathway first demonstrated in *E. coli*^5,14,15^ in which the free carboxyl group of malonyl-ACP is methylated by BioC, a SAM-dependent methyltransferase. Methylation masks the charge and tricks the fatty-acid biosynthetic enzymes into utilizing the malonyl-ACP methyl ester as a substrate (Fig. 1a). Following two cycles of standard fatty-acid synthesis reactions, the methyl group of the seven-carbon pimeloyl-ACP methyl ester is removed by BioH, a short-chain fatty-acid esterase, to give pimeloyl-ACP, which is condensed with l-alanine by BioF to generate 7-keto-8-aminopelargomic acid (KAPA, formal name 8-amino-7-oxononanoate), which begin assembly of the fused rings of biotin^5,14,15^.

**Fig. 2:**
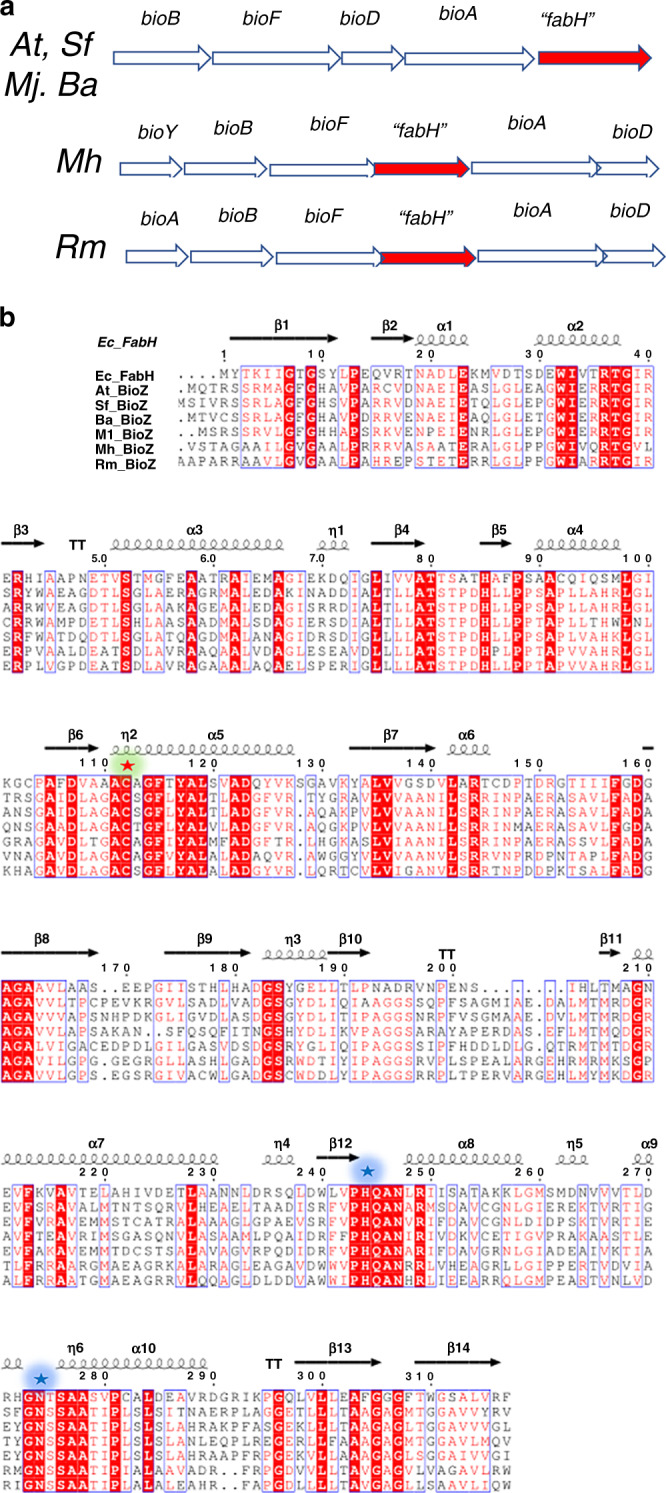
Biotin synthesis gene clusters containing putative *bioZ* genes and alignment of BioZ proteins with *E. coli* FabH. **a** The annotated biotin gene clusters of four species of α-proteobacteria are shown where “*fabH*” is *bioZ*. *At* is *Agrobacterium tumefaciens*, a plant pathogen; *Sf* is *Sinorhizobium fredii*, a plant symbiont; *Mj* is *Mesorhizobium japonicum* (Mj), formerly *Mesorhizobium loti* (Ml), another plant symbiont and *Ba* is *Brucella abortus*, an animal and human pathogen. Two thermophilic bacteria, *Marinithermus hydrothermalis* (*Mh*, an atypical *Bacteroidetes*) and *Rhodothermus marinus* (*Rm*), also contain *bioZ* (“*fabH*”) genes. **b** Multiple sequence alignments of *E. coli* FabH and the BioZ proteins. Amino-acid residues conserved between FabH and all BioZ proteins are in red. Potential BioZ catalytic residues conserved in KAS III proteins are highlighted in green (the active site cysteine residue that carries the acyl enzyme intermediate) or blue (the essential histidine and asparagine residues critical for the decarboxylation step of the FabH reaction).

The α-proteobacteria that are typified by the plant pathogen *Agrobacterium tumefaciens* and the mammalian pathogen *Brucella abortus* together with the symbiotic nitrogen-fixing bacteria *Mesorhizobium japonicum* and *Sinorhizobium fredii* synthesize the pimelate moiety by a distinctly different pathway. No genes encoding the known pimelate synthesis enzymes (BioC, BioH, BioW, or BioI) discussed above are found in these genomes. Instead, these bacteria have a gene encoding a putative 3-ketoacyl-ACP synthase (condensing enzyme-like) called *bioZ* clustered with the biotin ring-forming genes^16^ (Fig. 2a). BioZ proteins have ~35% sequence identity with the *E. coli* and *Streptomyces coelicolor* FabH 3-ketoacyl-ACP (acyl carrier protein) synthase III (KAS III) proteins, which catalyze the initial elongation/condensation in the fatty-acid synthetic pathway^17,18^ (Fig. 2b) and thus are usually annotated as FabH proteins.

FabH condenses a molecule of acetyl-CoA with a molecule of malonyl-ACP to form acetoacetyl-ACP and CO_2_^17,18^. After elimination of the resulting keto group and conversion to a butyryl-ACP primer, condensation with malonyl-ACP is catalyzed by the long chain ketoacyl-ACP synthases, FabB and FabF, resulting in synthesis of the acyl chains of the *E. coli* membrane lipids. FabH enzymes differ from the FabB–FabF class of enzymes in having a Cys-His-Asn catalytic triad rather than the Cys-His-His triad of FabB–FabF enzymes^19^. Two groups have proposed reaction mechanisms for FabH based on the *E. coli* FabH crystal structures. Although Qiu and colleagues obtained a more refined structure that showed the oxyanion hole and more detailed residue positioning^20^, the mechanism proposed by Davies and colleagues^21^ seems more in accord with the enzymological data.

In this work, we report that BioZ is the enzyme responsible for pimeloyl-thioester synthesis in α-proteobacteria. BioZ performs the FabH condensation reaction but uses glutaryl-CoA derived from lysine degradation as a primer for elongation with malonyl-ACP to give pimeloyl-ACP. *A. tumefaciens* mutant strains deleted for *bioZ* or that encode a mutant BioZ, lacking the key active site nucleophile require biotin for growth. Biotin synthesis is greatly and specifically stimulated by addition of lysine, glutarate or 2-oxohexanedioic acid (an intermediate in lysine degradation). Glutarate is converted to glutaryl-CoA by transfer of CoA from succinyl-CoA catalyzed by the type III acyl-CoA transferase, CaiB. Deletion of the *caiB* gene results in a biotin requirement for growth.

## Results

### The *bioZ* genes of diverse α-proteobacteria, an atypical Bacteriodes strain and a Thermus strain complement *E. colibioC* and *bioH* null mutant strains

In α-proteobacteria and a few other bacteria, *fabH* homologs are found within biotin synthesis gene clusters, whereas the canonical *fabH* genes are clustered with fatty acid and phospholipid synthesis genes. The biotin synthesis gene cluster FabH homologs were first reported by Ronson and coworkers^16^ who named these proteins BioZ. The amino-acid sequences of BioZ proteins indicate conservation of the canonical FabH Cys-His-Asn catalytic triad (Fig. 2b). These investigators^16^ confirmed the role of BioZ in biotin synthesis by inactivation of *bioZ* in *Mesorhizobium loti* sp. strain R7A (now *Mesorhizobium japonicum*^22^), which resulted in biotin auxotrophy. As the *M. japonicum* biotin gene cluster contained genes that replaced function of the *E. coli* enzymes required for assembly of the fused heterocyclic rings of biotin, BioZ seemed likely to function in the earliest phase of biotin synthesis, formation of the pimeloyl-thioester intermediate^16^. Moreover, *M. japonicum* lacks genes encoding homologs of the known pimeloyl-thioester synthesis enzymes suggesting that BioZ may replace these functions. Indeed, Ronson and coworkers reported that BioZ expression complemented growth of an *E. coli ∆bioH* strain^16^. However, these workers also reported that BioZ expression was unable to support growth of a *E. coli bioC23* mutant strain in the absence of biotin. This was puzzling because BioC and BioH act as partners (Fig. 1) in that BioC inserts the methyl group that must be removed by BioH when synthesis of the pimeloyl moiety is complete^5,14,15,23,24^.

**Fig. 3:**
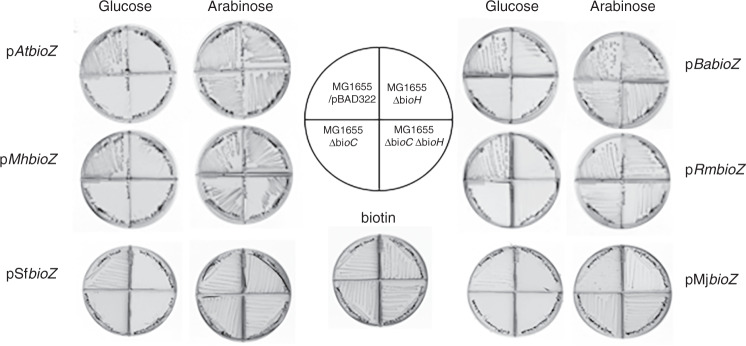
Diverse BioZ proteins complement *E. coli* strains having deletion alleles of *bioC*, *bioH,* or deletion alleles of both *bioC* and *bioH*. The α-proteobacterial bioZ genes were obtained by PCR amplification from the genomic DNAs and inserted into the arabinose expression vector pBAD322. The resulting plasmids were then transformed into the *E. coli* biotin auxotrophic strain and single transformant colonies were streaked onto biotin-free medium with either glucose or arabinose as sole carbon source as shown in the left and right columns of petri plates. The plates are divided into quadrants by plastic walls to prevent cross-feeding. Arabinose induces the pBAD promoter that drives BioZ expression, whereas glucose represses promoter activity. The plate below the plate-loading cartoon contained biotin. MG1655/pBAD322 denotes a wild-type strain carrying the empty arabinose vector plasmid. The PCR primers are given in Supplementary Table 1 and the strains are given in the Supplementary Table 2.

In prior work from this laboratory, *E. coli* biotin auxotrophs having clean in-frame deletion alleles of *bioH* and *bioC* were constructed^5^ and we used these strains to reassess BioZ function in *E. coli*. Six *bioZ* candidate genes and four *fabH* genes derived from α-proteobacteria plus the atypical Bacteroides and Thermus strains were tested. The genes were inserted into a plasmid vector under control of an arabinose regulated promoter and expressed in *E. coli bioH* and *bioC* deletion strains. Single colonies from the transformations were streaked on biotin-free M9 minimal media containing arabinose and an antibiotic selective for plasmid maintenance. All of the *bioZ* candidates complemented the *E. coli* ∆*bioC* and ∆*bioH* deletion strains as well as a ∆*bioC* ∆*bioH* strain deleted for both genes (Fig. 3). In contrast, the *fabH* genes encoded in the fatty-acid synthesis gene clusters of these organisms failed to complement any *bio* deletion strain (Supplementary Fig. 1b). These results indicate that the *bioZ* genes are involved in pimelate moiety synthesis, whereas the *fabH* genes play no role in biotin synthesis. The discrepancy between our ability to complement the ∆*bioC* and ∆*bioC ∆bioH* deletion strains with *bioZ* and the prior work, reporting an inability to complement the *bioC23* strain is unclear. The strain used by Ronson and coworkers was from a set of consecutively numbered biotin-requiring strains each lacking function of a known *bio* gene^16^. We obtained t6he R878 *bioC23* strain from the Coli Genetic Stock Center (Yale University) and found that introduction of plasmids encoding wild *M. japonicum bioZ* allowed growth in the absence of biotin (Supplementary Fig. 1a). Therefore, the most probable explanation for the failure of Ronson and coworkers^16^ to observe complementation was due to mislabeling or switching of the mutant strains such that the strain tested was not a *bioC* strain but rather a strain having a mutation in a different biotin synthetic gene. Indeed, the strain and allele numbers disagree with those given in the original paper^25^ consistent with a bookkeeping error.

### Deletion and active site mutants of *A. tumefaciens* bioZ are biotin auxotrophs

**Fig. 4:**
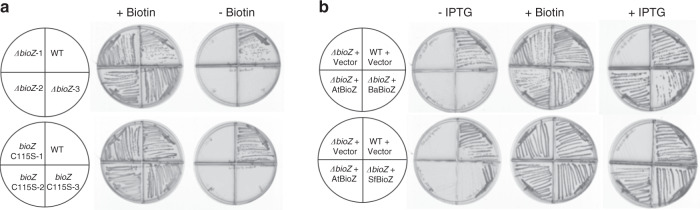
Deletion of *A. tumefaciens bioZ* or introduction of the BioZ C115S mutation resulted in biotin auxotrophy that was restored by expression of diverse BioZ proteins. **a** Three *∆bioZ* strains or three *bioZ* C115S strains were streaked on minimal medium plates that either contained or lacked biotin. **b** Complementation of a *A. tumefaciens ∆bioZ* strain by plasmids expressing the BioZ proteins of *A. tumefaciens (*At)*, B. abortus* (Ba) or *Sinorhizobium fredii* (Sf). IPTG was added to induce *bioZ* transcription.

Ronson and coworkers^16^ showed that the *M. japonicum* BioZ was required for biotin synthesis and argued that BioZ is involved in pimeloyl-thioester synthesis. To test if this was the case in another α-proteobacterium, we constructed both *bioZ* and *fabH* deletion mutant strains in the well-studied α-proteobacterium, *A. tumefaciens* C58 (recently renamed *A. fabrum* C58, but for consistency we shall retain the former name in this report), by homologous recombination. These strains were streaked on *Agrobacterium* minimal media with or without biotin supplementation. The ∆*bioZ* strains required biotin supplementation for growth (Fig. 4a), whereas the *fabH* disruption mutants lacked a growth phenotype (Supplementary Fig. 1c) These results confirm that *bioZ* is required for biotin synthesis in *A. tumefaciens,* whereas *fabH* has no role in this process. Plasmids encoding the *bioZ* or *fabH* genes from *A. tumefaciens* and other bacteria having a *bioZ* gene in their biotin clusters (Fig. 2) were constructed in the isopropyl β-d-1-thiogalactopyranoside (IPTG)-inducible pSRKGm vector to test for complementation of the *A. tumefaciens bioZ* deletion mutant strains. All of the *bioZ* genes tested complemented the *A. tumefaciens bioZ* deletion strain upon IPTG induction (Fig. 4b).

The reaction mechanism of FabH is conveniently divided into three steps: (i) acetyl transfer from acyl-CoA to the thiol of the active site cysteine 112, (ii) binding of malonyl-ACP via interactions of histidine 244 and asparagine 274 with the thioester carbonyl to generate and stabilize a carbanion with release of CO_2_, and (iii) the carbanion then performs nucleophilic attack of the acetyl-cysteine bond to form the elongated acyl chain^19^. To test if the *A. tumefaciens* BioZ cysteine residue C115 (analogous to *E. coli* FabH C112) has a catalytic role, C115 of the *A. tumefaciens* genomic *bioZ* gene was changed to a serine codon to give BioZ C115S. This mutation was also constructed in an AtBioZ expression plasmid for purification of the mutant protein. The *A. tumefaciens bioZ* C115S mutant strain required biotin for growth, indicating that C115 has a key role in catalysis (Fig. 4a).

### BioZ proteins catalyze condensation of glutaryl-CoA with malonyl-ACP to form pimeloyl-ACP

Upon expression in *E. coli* BioZ proteins showed generally poor expression with production of insoluble inclusion bodies, the only exception being *A. tumefaciens* BioZ, which was soluble but prone to aggregation. In attempts to cope with this shortcoming we expressed these *bioZ* genes in Rosetta pLysRARE (Novagen), which contains plasmids encoding the tRNAs that utilize codons rarely used in *E. coli*. We also used a synthetic version of *M. japonicum bioZ* gene composed of favored *E. coli* codons. However, upon expression of the other BioZ proteins gave only insoluble products. Thus, we used a modified heat-shock protocol for the these BioZ proteins and obtained soluble proteins presumably owing to induction of heat-shock chaperones that aided BioZ folding. Hence, all six BioZ proteins could be expressed and purified in soluble form (Supplementary Fig. 2).

**Fig. 5:**
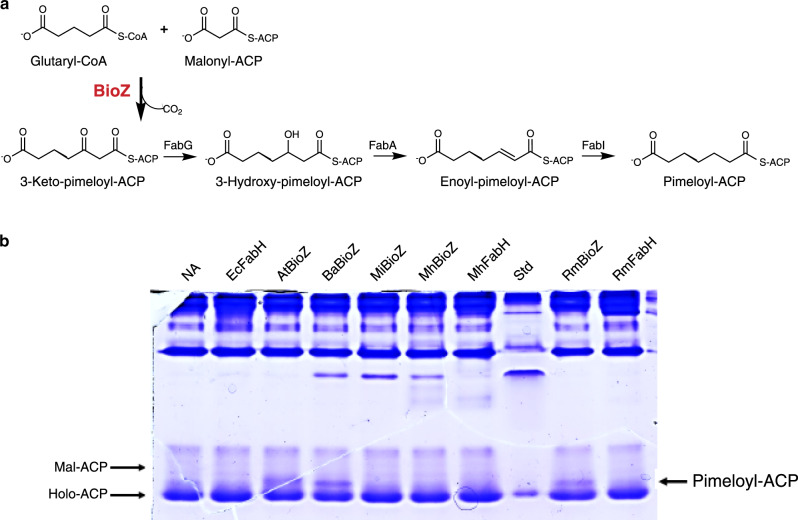
Condensation activity of BioZ. **a** Scheme for testing elongation of glutaryl-CoA with malonyl-ACP. The unstable product of decarboxylative condensation was converted to pimeloyl-ACP by successive reduction, dehydration, and reduction steps catalyzed by the *E. coli* fatty-acid synthetic (Fab) enzymes as shown. BioZ is in red font. **b** Conformationally sensitive urea electrophoretic separation of ACP species. The At, Ba, and Rm BioZ proteins catalyze formation of a product having the mobility of pimeloyl-ACP whereas three different FabH proteins and the Mj and Mh BioZs failed to form the product. This gel and gels in which alternate primers and elongation substrates were tested are given together in Supplementary Figure 3. Each experiment was repeated more than three times and the gel given is representative. Strain abbreviations are given as in Fig. 4.

Given the highly purified proteins we proceeded to establish the substrates of the BioZ reaction. Sequence alignments and the dependence of in vivo activity on residue C115 strongly argued that BioZ would catalyze a FabH-like reaction. FabH utilizes a CoA thioester (acetyl, propionyl, butyryl) as a substrate, which after transfer of the acyl group to the active site cysteine becomes extended by decarboxylative condensation with malonyl-ACP^19^. Hence, the most straightforward route of pimelate thioester synthesis in *A. tumefaciens* would be for BioZ to use a CoA ester as a substrate and transfer the acyl group to the thiol of the active site cysteine for extension by condensation with malonyl-ACP (or perhaps malonyl-CoA). As BioZ expression in *E. coli* bypassed the need for BioC catalyzed methylation, we assumed that BioZ could accommodate a substrate having a free carboxyl group. Given this assumption and the specificity of FabH for CoA thioesters, we tested malonyl-CoA and glutaryl-CoA as BioZ substrates. As 3-ketoacyl-thioesters are unstable, the reactions contained the purified *E. coli* fatty-acid synthetic enzymes; 3-ketoacyl-ACP reductase (FabG), 3-hydroxyacyl ACP dehydratase (FabA), and enoyl-ACP reductase (FabI) and NADH to convert the condensation product to the stable fully saturated form. By use of conformationally sensitive urea-PAGE gels, we found that BioZ condensed glutaryl-CoA with malonyl-ACP to give a product that had the gel mobility of pimeloyl-ACP (Fig. 5). The product, which migrated just behind holo-ACP, as previously observed for pimeloyl-ACP^5,14^, was formed when the reactions contained BioZ proteins from *A. tumefaciens, B. abortus,* or *R. marinus* (Fig. 5). Three FabH enzymes included as controls failed to produce this species. Two BioZ proteins, those from *M. japonicum* and *M. hydrothermalis*, were inactive, suggesting inactivation during purification or inhibition by bound heat-shock chaperones (Supplementary Fig. 3). It is also possible that the assay temperature was too low for the thermophile protein (higher temperatures would inactivate the fatty-acid synthetic proteins). When malonyl-CoA was the primer no product was detected (Supplementary Fig. 3).

**Fig. 6:**
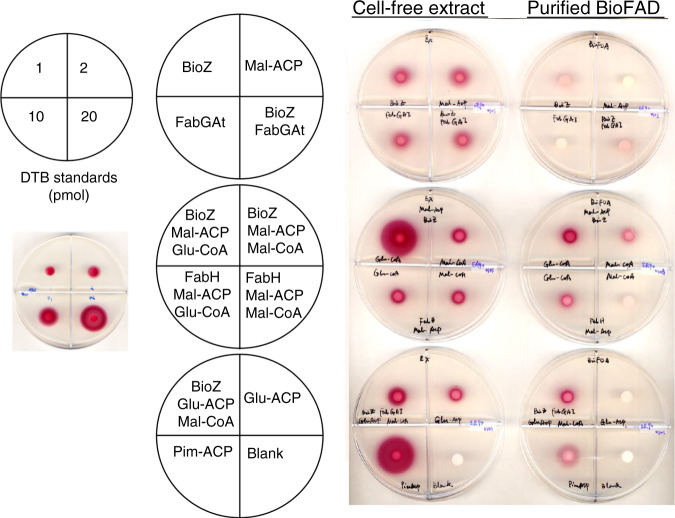
Functional replacement of *E. coli* BioC and BioH with AtBioZ in vitro. The preparations tested were either a fractionated and dialyzed extract of the *E. coli ∆bioC ∆bioH* strain STL25 or a mixture of purified *E. coli* DTB synthesis proteins, BioF, BioA, and BioD as depicted above the plates. The components in each of the reactions applied to the bioassay plates are given in the plate depictions to the left of the cell-free extract assays. The contents of the reactions containing BioF, BioA, and BioD and their substrates are given to the right of those assay plates. Response to DTB standards is given in the box on left side of the figure. The top plates contain controls. In a more-defined assay, we reconstituted DTB synthesis in vitro using BioZ proteins and the purified *E. coli* biotin ring synthesis enzymes, BioF, BioA, and BioD, plus the substrates required for their reactions. As purified BioB is inactive under aerobic conditions, it was omitted and thus the assayed product was DTB. Although the DTB production was low owing to the weak activity of the purified BioD and the relative insensitivity of DTB bioassay (five to sixfold less sensitive than is the biotin bioassay) these results showed that BioZ proteins use glutaryl-CoA and malonyl-ACP as substrates to synthesize the pimeloyl-ACP required for DTB synthesis. To a much lesser extent, the combination of glutaryl-ACP and malonyl-CoA was also active (Fig. 6).

To verify that the product of these reactions was pimeloyl-ACP, the biotin pathway was reconstituted in vitro using purified BioZ proteins and cell-free extracts of an *E. coli* ∆*bioC* ∆*bioH* mutant strain plus glutaryl-CoA and malonyl-ACP. The reactions were assayed for production of biotin and/or dethiobiotin (DTB) using the *E. coli* biotin auxotrophic strain ER90, which responds only to biotin and its immediate precursor, DTB. Biotin/DTB synthesis was detected when glutaryl-CoA and malonyl-ACP were added to the enzyme reaction mixture, indicating that BioZ proteins condense a glutaryl moiety with a decarboxylated malonyl moiety to make the pimeloyl precursor required for biotin synthesis. In contrast, only low levels of biotin/DTB production was detected when using glutaryl-ACP and malonyl-CoA as substrates (Fig. 6).

Previous studies have shown that *B. subtilis* BioF specifically accepts only pimeloyl-CoA as acyl donor, whereas pimeloyl-ACP is inactive both in vivo and in vitro^12^. In contrast, *E. coli* BioF accepts either of the pimeloyl-thioesters, although pimeloyl-ACP is strongly favored. To determine whether the pimeloyl moiety formed by in vitro BioZ reactions is attached to CoA or to ACP, either *B. subtilis* BioF or *E. coli* BioF was used for in vitro pathway reconstitutions. KAPA synthesis was detected by bioassay using an *E. coli ∆bioF* strain. KAPA formation was detected only in the reactions that contained *E. coli* BioF, indicating that the in vitro BioZ reaction product is pimeloyl-ACP. Several BioZ proteins were used to test if they catalyze the same reaction with two sets of substrates: malonyl-ACP and glutaryl-CoA or glutaryl-ACP and malonyl-CoA. These data suggest that pimeloyl-ACP rather than pimeloyl-CoA is produced by all BioZs. Although the BioZs from other microorganisms seems to have higher activity on glutaryl-ACP than does AtBioZ (Supplementary Fig. 4). However, as glutaryl-ACP has yet to be observed in nature, these observations seem to lack biological relevance. Note that CoA and ACP substrates often mimic one another (there are many examples in fatty-acid synthesis) with the noncognate substrate giving only weak activity. CoA and ACP thioesters share long flexible 4’-phosphopanthetine arms that carry the thioester and both are acidic molecules. Moreover, many enzymes in fatty acid and polyketide synthesis can use thioesters of *N*-acetylcysteamine, which is composed of only the thiol proximal segment of the 4’-phosphopanthetine moiety.

**Fig. 7:**
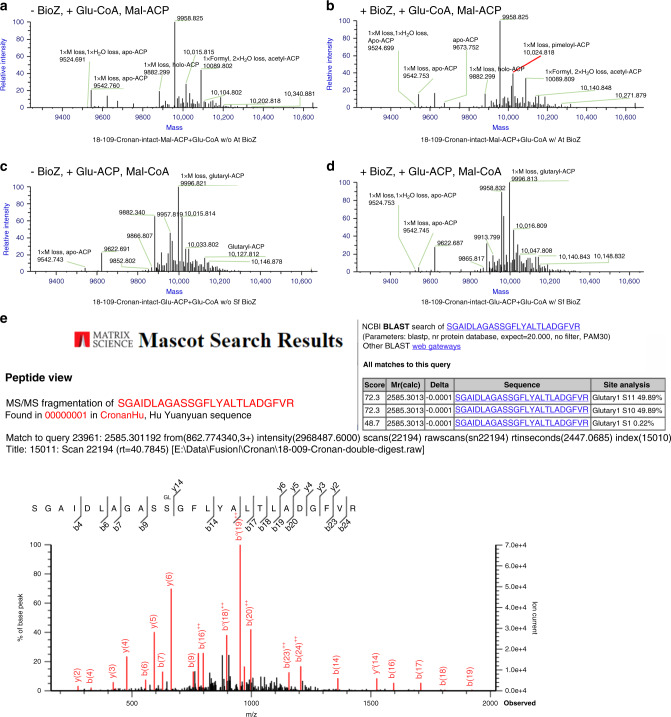
Mass spectral analysis of pimeloyl-ACP synthesis and MS/MS detection of a trapped  active site glutarate adduct. Mass spectral analyses of intact ACP species **a**–**d** and of the modified active site peptide of the BioZ C115S mutant protein **e**. **a** Reaction containing malonyl-ACP and glutaryl-CoA as substrates and lacking AtBioZ **b**. Reaction containing malonyl-ACP and glutaryl-CoA as substrates supplemented with AtBioZ. **c** and **d** tested glutaryl-ACP and malonyl-CoA as substrates with or without SfBioZ addition. **e** MS/MS spectra of the active site peptide of the AtBioZ C115S purified from an *E. coli* expression strain. Analysis by the MASCOT program (inset at top right) indicated the presence of glutaryl modifications of serine residues 115 and 116. Traces of pimeloyl modification were also present whereas no malonyl modification was seen.

To confirm the synthesis of pimeloyl-ACP in in vitro BioZ reactions and to test possible relief of product inhibition by addition of the downstream enzymes (Fig. 6 and Supplementary Fig. 4), MALDI-mass spectrometry of the intact protein was used to analyze the acyl-ACP species formed. We synthesized the substrates using a Strep-tagged ACP that allowed use of a Strep-Tactin column for removal of the minor contaminants present in ACP purified by the usual ion exchange chromatography step^26^. Following reaction completion both the substrate and product ACP species were again purified using a Strep-Tactin column. Using Thermo Q Exactive HF-X Hybrid Quadrupole-Orbitrap Mass Spectrometry, we detected BioZ-catalyzed synthesis of pimeloyl-ACP from the in vitro reactions using malonyl-ACP and glutaryl-CoA (Fig. 7a–d). Note that upon overexpression of *E. coli* ACP a portion of the protein retains the N-terminal methionine^27^ owing to titration of methionine aminopeptidase which complicates the spectra (both proteins are fully active).

Without addition of AtBioZ the reaction containing malonyl-ACP and glutaryl-CoA results in mainly acetyl-ACP with a mass of 10,089 m/z. which arises by decarboxylation of the unstable malonyl-ACP, with a formyl modification and water loss. Apo-ACP and holo-ACP lacking the N-terminal methionine (annotated as 1xM loss) are also detected (Fig. 7a). In the reaction containing active AtBioZ, a mass of 10,024 indicating pimeloyl-ACP with a lack of the N-terminal methionine has been detected in the mass spectra (annotated as 1xM loss, pimeloyl-ACP in red). Similar peaks of acetyl-ACP, apo and holo-ACP were also detected (Fig. 7b).

Note that AtBioZ and SfBioZ share the highest sequence identity at ~70%. SfBioZ was observed to have greater activity than AtBioZ with glutaryl-ACP and malonyl-CoA in Fig S4. However, a peak calculated as pimeloyl-ACP was not detected in the reaction with or without active SfBioZ (Fig. 7c, d). This suggested that malonyl-ACP and glutaryl-CoA comprise the preferred substrate combination for BioZ. As far as we know, there is no evidence that intracellular glutaryl-ACP exists. On the other hand, malonyl-ACP is a common ACP species that is a well-known substrate of fatty-acid synthesis. Therefore, malonyl-ACP and glutaryl-CoA are the most likely intracellular BioZ substrates.

### Evidence that glutaryl-CoA is the physiological BioZ primer substrate

As discussed above, the FabH reaction proceeds through formation of an enzyme bound intermediate in which the primer becomes covalently bound to the thiol of the active site cysteine via a thioester linkage. We hypothesized that conversion of the cysteine to a serine might result in trapping of the acyl enzyme intermediate because the less-reactive oxygen ester would be formed which would be a poor substrate for nucleophilic attack by the carbanion derived from malonyl-ACP. A similar substitution of serine for the active site cysteine residue of the 3-ketoacyl-ACP synthase domain of the polyfunctional mammalian fatty-acid synthase showed formation of acetyl oxygen esters^28^. We therefore expressed the completely inactive AtBioZ C115S protein in *E. coli* in hopes of detecting a trapped acyl enzyme intermediate.

In contrast to the poorly soluble wild-type protein, the C115S protein was very soluble upon expression in *E. coli*. Tandem mass spectroscopy of the active site peptide (residues 106–160) obtained by trypsin digestion of the mutant protein showed that the serine residue that replaced C115 had a significant glutaryl modification, whereas no malonyl modification and only a trace of pimeloyl modification were present (Fig. 7e). Note that S116 also carried a glutaryl moiety. As this residue is next to Ser115 these glutaryl moieties seem likely to have arisen by chemical transesterification from S115. The glutaryl modifications, which must have been formed from glutaryl-CoA present in the *E. coli* cytosol, indicate that glutaryl-CoA is the physiological substrate that acylates the active site cysteine residue of BioZ.

### Resting cells of *A. tumefaciens* synthesize the biotin pimelate precursor from l-lysine, glutaric acid, or 2-oxoadipate

**Fig. 8:**
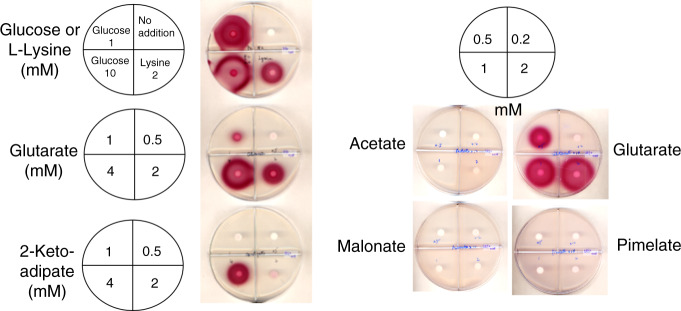
Resting cells of the *A. tumefaciens* Δ*bioR* mutant strain synthesize biotin when supplemented with l-lysine, 2-oxoadipate, or glutarate. No detectable biotin was synthesized by the Δ*bioR* strain when acetate, malonate, or pimelate were the supplements. The sectors were divided by plastic walls to prevent cross-feeding and the compound incubated with each resting cell sample and their concentration are given on the Figure. Note that addition of glucose allows the resting cells to resume growth. Resting cells were used to avoid the complication of competing reactions and substrates during growth.

The in vitro experiments above indicated that glutaryl-CoA is a bona fide intermediate in *A. tumefaciens* biotin synthesis. However, the source of glutaryl-CoA was unknown. Fortunately, two brief papers appeared in the early 1970s in which Ogata and coworkers^29,30^ reported that that *Agrobacterium radiobacter* IAM 1562 and related bacteria synthesized DTB from glutarate in resting cell suspensions. l-Lysine supplementation also stimulated DTB production, although the levels obtained were about one-fourth those obtained with glutarate. Glutarate was further implicated by incorporation of radioactive glutarate into DTB^30^ (the labeled DTB carbon atoms were not determined). These publications implicated lysine degradation as the source of glutarate for DTB synthesis. We extended this work in an *A. tumefaciens bioR* deletion mutant which excretes virtually all of its biotin into the medium^31^. Biotin synthesis from exogenous dicarboxylic acids was tested as in the prior work using modified resting cells preparations. After preparation of the resting cell suspensions in minimal medium lacking a carbon source, various dicarboxylic acids were added followed by incubation for 12–16 h. The culture media were concentrated tenfold and spotted on plates containing strain *E. coli* ER90 to assay for biotin/DTB production. Unlike *B. subtilis* which directly incorporates exogenous pimelate into biotin^6,12^, *A. tumefaciens* failed to incorporate pimelate into biotin/DTB but rather used glutarate or compounds readily converted to glutarate to synthesize the pimelate precursor (Fig. 8). No biotin/DTB was formed in resting cell suspensions supplemented with acetate, malonate, succinate or pimelate. The levels of biotin/DTB produced depended on the concentration of glutaric acid in accordance the reports of Ogata and coworkers^29,30^. *A. tumefaciens* resting cell suspensions also produced biotin/DTB when supplemented with l-lysine or with the lysine degradation intermediate, 2-oxoadipate (2-oxohexanedioic acid) (Fig. 8). However, in a recent study on *Pseudomonas putida* KT2440 lysine degradation, 2-oxoadipate is an intermediate in D-lysine degradation that is converted to the TCA cycle intermediate 2-ketoglutarate^32^.

### A key enzyme in l-lysine degradation provides glutaryl-CoA for biotin synthesis

**Fig. 9:**
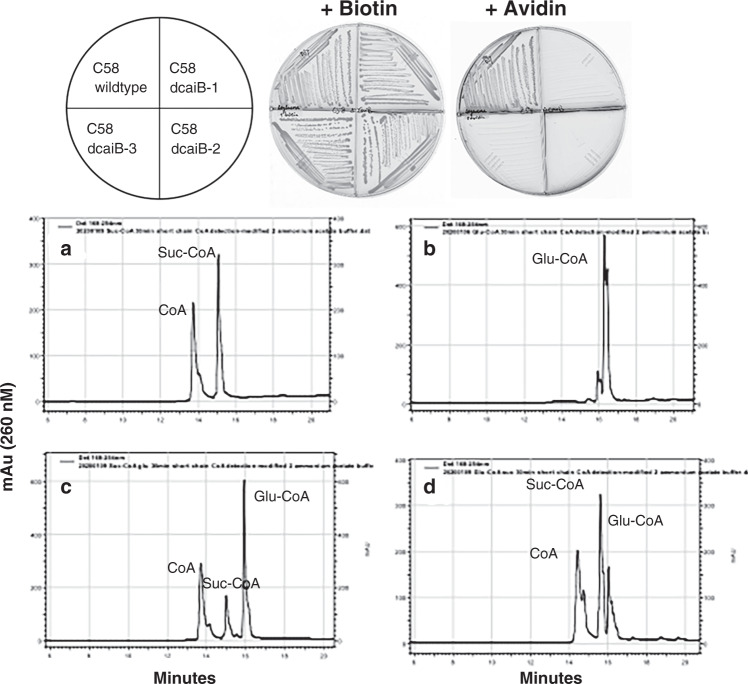
*A. tumefaciens* CaiB is essential for biotin synthesis and catalyzes transfer of CoA from succinyl-CoA to glutarate to produce glutaryl-CoA. **a** Growth of the *A. tumefaciens ∆caiB* strain on minimal medium containing either biotin or the high-affinity biotin binding protein, avidin. Three mutant colonies were streaked. **b**–**e** HPLC analyses of CaiB activity by reverse phase HPLC monitored at 260 nm. **b** succinyl-CoA standard; **c** glutaryl-CoA standard. **d** Transfer of CoA from succinyl-CoA to glutarate and **e** transfer of CoA from glutaryl-CoA to succinate. Note that succinyl-CoA is unstable in the assay and gives rise to free CoA.

The *A. tumefaciens* C58 Atu2127 gene encodes a predicted type III acyl-CoA transferase (annotated as CaiB) that seemed a likely candidate for conversion of glutarate to glutaryl-CoA via transfer of CoA from succinyl-CoA. The possibility that CaiB might provide glutaryl-CoA for the BioZ reaction was tested by disruption of the *caiB* gene. Loss of CaiB activity should result in biotin auxotrophy and this was the case (Fig. 9a). After construction and testing the *caiB* disruption strain, two reports on l-lysine degradation in *Pseudomonas putida* KT2440 appeared. These reports showed this bacterium has two pathways for lysine degradation, a glutaryl-CoA-dependent pathway and a second pathway involving L-2-hydroxyglutarate^32,33^. Blocking the glutaryl-CoA-dependent pathway prevented growth on glutarate, whereas growth on l-lysine was normal^33^. Expression of a *P. putida* CaiB homolog called GcdG that is 56% identical to the *A. tumefaciens* C58 CaiB was induced upon growth with l-lysine or glutarate thereby implicating this enzyme in lysine degradation. However, neither GcdG enzyme activity nor *gcdG* gene inactivation were reported. The *A. tumefaciens* C58 genome lacks recognizable genes encoding key enzymes of the L-2-hydroxyglutarate pathway of lysine degradation and thus the glutaryl-CoA route may be the sole l-lysine degradation pathway. To test the proposed enzymatic activity of the *A. tumefaciens* C58 Atu2127 gene product we purified the protein to homogeneity (Supplementary Fig. 5) and tested its ability to transfer CoA from succinyl-CoA to glutarate (Fig. 9). As expected from studies of other type III acyl-CoA transferases^34^, the reaction was reversible. The *A. tumefaciens* C58 Atu2127 protein could also utilize acetyl-CoA in place of succinyl-CoA. Further characterization of this enzyme will be reported elsewhere.

## Discussion

Pimeloyl-ACP synthesis in *A. tumefaciens* and other α-proteobacteria uses an unusual primer substrate, glutaryl-CoA. Since glutaryl-CoA is generally associated with degradative pathways such as that for lysine^32^ this raises the question of how an essential pathway, biotin synthesis, can depend on a degradative pathway such as lysine catabolism. Two factors provide a rationale. First, *A. tumefaciens* like most bacteria requires only trace amounts of biotin. *A. tumefaciens* biotin auxotrophs grow well when supplemented with 2 nM biotin, a concentration essentially identical to the requirement of *E. coli* biotin auxotrophs^31^. Since *E. coli* requires only a few hundred biotin molecules per cell^35^, *A. tumefaciens* seems likely to require a similarly small number of biotin molecules. A second factor is that lysine is an abundant protein residue and *A. tumefaciens* and other plant associated α-proteobacteria contain a lysine-modified phospholipid^36^. Hence glutaryl-CoA resulting from degradation of lysine liberated in turnover of proteins and phospholipids could readily provide the modest glutaryl-CoA levels required for pimeloyl-ACP synthesis. A recent example of a degradative pathway involved in synthesis of a cofactor is synthesis of the pantothenate precursor, β-alanine by degradation of uracil^37^.

The ability of BioZ to functionally replace BioC and BioH in *E. coli* demonstrates that BioZ is responsible for synthesis of the pimeloyl-ACP required for assembly of the biotin heterocyclic rings. BioZ is a homolog of FabH modified to allow a longer chain primer, glutaryl-CoA having a charged ω-carboxyl group, to access the active site cysteine thiol. We expect that relative to *E. coli* FabH, BioZ accommodates glutaryl-CoA by expanding the size of the primer binding pocket and by neutralizing the charge of the free glutaryl-CoA carboxyl with a basic amino-acid sidechain at the distal end of the pocket. An arginine sidechain seems a likely candidate to interact with the free carboxyl group as is the case with malonyl-CoA: ACP transacylase^38^.

Note that BioZ is not the first FabH hetero-functional FabH homolog to utilize a amino-acid-related primer. The PqsD protein of *Pseudomonas aeruginosa*, which is 39% identical to *E. coli* FabH with the same catalytic triad, catalyzes the condensation of the CoA ester of anthranilic acid, a tryptophan synthetic intermediate, with malonyl-ACP (or CoA)^39^. The anthranilic acid moiety (essentially benzoic acid having a single amino substituent) is utilized by PqsD despite its size, planar ring and amino group. Notably the α-carbons of the crystal structures of PqsD and *E. coli* FabH can be aligned within 1.4 Å over the length of FabH (PqsD is twenty residues longer). This structural conservation is striking given the very different substrates (acetyl-CoA versus anthraniloyl-CoA) and strongly suggests that PqsD must undergo very significant structural conformations. The *Mycobacterium tuberculosis* FabH is also thought to undergo large conformation changes that allow it to accommodate acyl-CoAs having a wide spectrum of acyl chain lengths (C6 to C 20)^40^. In addition to PsqD the FabH scaffold seems able to accommodate a wide variety of primers since there are other FabH-like proteins that are not involved in fatty-acid synthesis^41,42^. Examples are the archaeal proteins often annotated as FabH homologs (e.g., microbesonline.org) although the archaea do not synthesize fatty acids. These proteins are probably 3-hydroxy-3-methylglutaryl CoA synthases involved in assembly of the isoprenoid lipids that comprise the archaeal cell membranes. If so, these enzymes would use an acetoacetyl primer^43^.

## Methods

### Media

LB medium was used as the rich grow media for *E. coli* and *A. tumefaciens* strains. The *E. coli* minimal medium contained M9 minimal salts supplemented (final concentrations) with 0.2% (w/v) glucose, 0.1% (w/v) Vitamin Assay Casamino acids (Difco), 1 mM MgSO_4_, and 1 μg/ml thiamine, except when 0.2% (w/v) l-arabinose or a mixture of l-arabinose and glycerol replaced glucose for induction of the pBAD promotor. The *Agrobacterium* minimal medium^44^ consists of 1× AT buffer, which contains 3.95 mM KH_2_PO_4_, adjusted to pH 7 with NaOH, 1× AT salts, which contains 0.75 mM (NH_4_)_2_SO_4_, 30 μM MgSO_4_·7H_2_O, 3 μM CaCl_2_·2 H_2_O, 0.355 μM MnSO_4_· H_2_O, and 1× iron stock containing 2.5 mM, FeSO_4_·7 H_2_O, and 0.2% mannitol, or glucose as carbon source.

Genetic complementation experiments requiring expression of pBAD (for *E. coli*) and pSRK (for *A. tumefaciens*) promoters used minimal media containing either 0.2% arabinose or 1 mM isopropyl β-d-1-thiogalactopyranoside for induction, respectively. Antibiotics were utilized in the following concentrations (in µg/ml): sodium ampicillin (100), kanamycin sulfate (50) for *E. coli* or (100) for *A. tumefaciens*, chloramphenicol (20), gentamycin sulfate (20), streptomycin sulfate (100), and spectinomycin sulfate (50). Petri plates divided into sectors by plastic walls were used to prevent cross-feeding.

### Plasmids (Supplementary Table 2) and PCR primers (Supplementary Table 1)

Complementation plasmids (Supplementary Table 2) were constructed using pBAD322C^45^ for *E. coli* or pSRKGm^46^ for *A. tumefaciens*. The PCR primers used are given in Supplementary Table 1. Protein expression plasmids were constructed using pET28b or pET30a plasmids and transformed into either BL21(DE3) or Rosetta pLysRARE (Novagen). Most of the expression plasmids were constructed with N-terminal hexahistidine-tags. The exception is native ACP, which was not tagged and the ACP protein used in mass spectral experiments, which was fused with a strepavidin-tag with the amino-acid sequence Trp-Ser-His-Pro-Gln-Phe-Glu-Lys on the C-terminus.

*A. tumefaciens* deletion strains were constructed in pK19*mobsacB*^47^ for replacement of chromosomal copies by homologous recombination. First crossover colonies in which the plasmid construct had integrated into the chromosome were selected by antibiotic resistance, whereas second crossover events were obtained by counter-selection on LB containing 10% sucrose.

### Expression and purification of proteins

Unless specifically mentioned below, strains expressing hexahistidine-tagged proteins were grown in LB media containing the appropriate antibiotics at 37 °C until OD_600_ reached 0.8–1, then were induced for overexpression with 0.5 mM IPTG for 3–4 h at 37 °C. A Beckman-Coulter AKTA FPLC purification system and GE HisTrap HP His-tag purification columns were used to purify proteins with Buffer A containing 50 mM sodium phosphate, pH8.0, 500 mM sodium chloride, and Buffer B containing 50 mM sodium phosphate (pH 8.0), 500 mM sodium chloride and 500 mM imidazole for elution. Cells were collected from 1000 ml of culture by centrifugation, suspended in 10 ml of buffer A with addition of 10 mg lysosome at 37 °C. Cells were lysed by three passages through a French pressure cell. Supernatants obtained after centrifugation at 40,000 × *g* for 30 min were injected into the AKTA FPLC system and washed with 100 ml of Buffer A and 50 ml of 4% Buffer B. The proteins were eluded with 50 ml of each 8%, 16%, 50%, and 100% Buffer B. Fractionations of elution were collected every 12.5 ml and samples of the fractionations were loaded on sodium dodecyl sulfate polyacrylamide gel electrophoresis (SDS-PAGE) in order to determine protein purity (Supplementary Fig. 2). The fractions having pure expressed proteins were concentrated by using spin columns with 10 kDa cutoff membrane and centrifuge at 3700 × *g* then dialyzed in Buffer A at 4 °C overnight to remove imidazole.

### Heat-shock and osmotic stress treatments for expression of soluble BioZ proteins

The heat-shock and osmotic stress treatment was modified from that reported previously^48^ for the expression of soluble BioZ protein. In brief, cultures were inoculated and grown overnight in 20 ml LB medium with the appropriate antibiotics, then transferred into 1.8 liter of LB medium with appropriate antibiotics. When OD_600_ reached 0.8–1, the culture was incubated in a 50 °C water bath for 30 min, then cooled to room temperature. Final concentrations of 0.2% glucose and 10% glycerol were then added to the medium before induction with 0.1 mM IPTG overnight at 18 °C. Protein purifications were performed as described above using Beckman-Coulter AKTA FPLC purification system and GE HisTrap HP His-tag purification columns. Eluted fractions were collected every 12.5 ml and samples of the fractions were loaded on SDS-PAGE gels in order to determine protein purity. The fractions having highly purified protein were concentrated by using spin columns with 10 kDa cutoff membrane and centrifugation at 4000 × *g*, then dialyzed against Buffer A at 4 °C overnight to remove imidazole. BioZ proteins co-purified with several heat-shock proteins. Attempts to removing these proteins resulted in protein aggregation and denaturation. Therefore, the concentration of BioZ proteins were determined by calculating the fraction of BioZ protein bands on the SDS gels.

### Purification of *E. coli* holo-ACP and apo-ACP

*E. coli* holo-ACP and apo-ACP were obtained as previously described^49^. In brief, strain DK754 containing pJT93 (encoding *acpS* for preparation of holo-ACP) or pJT94 (encoding *acpH* for preparation of apo-ACP) were inoculated in 10 ml LB medium containing kanamycin, chloramphenicol, and spectinomycin and grown at 37 °C overnight. The starter cultures were transferred into 1 L LB medium containing the same antibiotics and allowed to grow until OD_600_ reached 0.8–1 at 37 °C. Coexpression of ACP with either the AcpS 4’-phosphopantetheinyl transferase or with the AcpH ACP phosphodiesterase were induced by addition of 0.2 mM IPTG followed by incubation at 37 °C for 4 h. Cells were harvested and washed in 10 ml of 50 mM Tris-HCl (pH 8.8). The cells were then resuspended in 10 ml reaction buffer that contained 50 mM Tris-HCl (pH 8.8), 10 mM MgCl_2_, 5 mM dithiothreitol, and lysed either by sonication or by three passages through a French pressure cell. The lysates were then cleared by centrifugation at 39,000 × *g* for 20 min at 4 °C, then incubated at 37 °C for 4 h with or without 1 mM CoA for preparation of holo-ACP or apo-ACP, respectively. Purification of ACP species was performed as previously described^49^.

### Enzymatic synthesis, purification, and visualization of ACP species

All acyl-ACP substrates were prepared from *E. coli* apo-ACP or holo-ACP. Acetyl-ACP, malonyl-ACP, and glutaryl-ACP were synthesized from apo-ACP and the appropriate short-chain acyl-CoA using *B. subtilis* Sfp 4’-phosphopantetheinyl transferase^49^, then purified. In brief, the reaction contained 100 mM potassium phosphate buffer (pH 7), 20 mM MgCl_2_, 0.5 mM dithiothreitol or (tris(2-carboxyethyl)phosphine) (TCEP), 0.75 mM acyl-CoA, 0.5 mM apo-ACP, and 10 μM Sfp. The reactions were incubated at 37 °C for 4 h. Acyl-ACPs were purified by ion exchange chromatography using Vivapure D spin columns (GE Healthcare Life Sciences). The reaction mixtures were loaded in a binding buffer containing 25 mM 4-morpholineethanesulfonic acid, pH 6, and 1 mM dithiothreitol. The column was washed with binding buffer containing 100 mM LiCl, then 250 mM LiCl. ACP species were eluted in binding buffer containing 500 mM LiCl, desalted, and analyzed in a conformationally sensitive electrophoretic mobility assay^49^ in 20% polyacrylamide gels containing 1 M urea at 100 V for 2 h. The proteins were then visualized by staining the gels with a solution of 50% methanol, 10% acetic acid, and 0.1% Coomassie Brilliant Blue R250 followed by destaining in 10% methanol and 10% acetic acid.

Pimelic acid is not a substrate for AasS^5^ and hence pimeloyl-ACP was synthesized via AasS-catalyzed thioesterification of holo-ACP with pimelate methyl ester^5,50^ followed by BioH-catalyzed cleavage of the methyl ester moiety^5,14^. In brief, the reaction contained 50 mM Tris-HCl (pH 8.5), 20 mM MgCl_2_, 0.5 mM dithiothreitol or TCEP, 5 mM ATP, 2.5 mM pimelate methyl ester, 0.5 mM holo-ACP. and 10 μM AasS. The reactions were incubated at 37 °C for 3 h. Pimeloyl-ACP was obtained by hydrolysis of the methyl group by addition of 10 μM BioH and incubation for 3 h at 37 °C. Purification and visualization of pimeloyl-ACP was performed as described above.

### Preparation of cell-free extracts

Preparation of strain STL96 (MG1655 *ΔbioC ΔbioH*) cell-free extracts carrying the *ΔbioC bio* operon plasmid, pCY123) was done as previously described^5^. Strain STL96 was grown at 37 °C to OD_600_ 0.8 in 250 ml M9 minimal medium containing 2 nM biotin. The cells were washed with M9 salts medium to remove biotin and subcultured into 1 L of glucose M9 minimal medium at for 6 h at 37 °C to derepress *bio* operon transcription by starvation for biotin. The cells were lysed in assay buffer containing 50 mM Na-MOPS and 200 mM KCl by three passages through a French Pressure cell. The lysate was then centrifuged at 20,000 × *g* for 20 min to obtain the soluble fraction. Ammonium sulfate was slowly added to the supernatant to 85% of saturation on ice under constant stirring until completely dissolved. The protein precipitant was collected by centrifugation at 10,000 × *g* and stored at −80 °C. The precipitate was solubilized before use by dialysis using 7000 kDa molecular weight cutoff membranes against assay buffer at 4 °C for 4 h to remove ammonium sulfate and any remaining small molecules.

### Preparation of bioassay plates and assays for KAPA, DTB, and biotin

*E. coli* strains ER90 (*ΔbioF bioC bioD*), NRD25 (MC1061 *∆bioABFCD*) and STL108 (MG1655 *ΔbioF ΔbioH*::Km) were used as assay organisms to detect the presence of DTB/biotin, biotin or KAPA, respectively. Cultures were grown in 5 ml of glucose M9 minimal medium containing 2 nM biotin at 30 °C overnight. The cells were harvested, washed with M9 medium and subcultured in 100 ml of minimal medium at 37 °C for 6 h to starve the cells for biotin. Avidin (0.1 units/ml) was added to the medium to prevent cross-feeding and neutralize any biotin or DTB contamination. The cells were collected by centrifugation, washed again in M9 medium to remove avidin and mixed with 150 ml of melted glucose minimal agar containing the redox indicator 2,3,5-triphenyl tetrazolium chloride (0.1%, w/v). The final OD at 600 nm was ~0.1. Five mL of this mixture was poured into Petri dishes sectored with plastic walls to prevent cross-feeding. A 6 mm diameter paper disk (BBL) was placed upon the agar of a sector and the disk was spotted with 10 μl of a reaction to be tested followed by incubation of the plates at 30 °C overnight. In the bioassay the test samples diffuse from the filter disks placed on the agar surface into agar seeded with strain ER90/ NRD25/ STL108. If growth occurs, the redox indicator 2,3,5-triphenyl tetrazolium chloride in the agar becomes reduced by cell metabolism to form a bright red, insoluble formazan deposit whose area is proportional to the concentration of the biotin pathway intermediate^51^. Quantification of biotin or DTB synthesis was done by comparison with dilutions of authentic standards.

### In vitro synthesis of DTB, biotin or KAPA

In vitro DTB/biotin synthesis. This assay allows in vitro conversion of ACP-bound substrate into DTB or biotin using either the enzymes of cell-free extracts or purified DTB synthesis enzymes. A 100 μl reaction in assay buffer contained 2.5 mg cell-free extract protein or 1 mM each of purified *E. coli* BioF, BioA, and BioD in assay buffer was performed in 1 μmol MgCl_2_, 0.5 μmol dithiothreitol, 0.01 μmol pyridoxal-5′-phosphate, 50 μg malonyl-ACP, 1 μM BioZ, FabG, FabA, FabI, 0.1 μmol l-alanine, 0.1 μmol KHCO_3_, 0.1 μmol NADPH, 0.1 μmol ATP, 0.1 μmol glucose-6-phosphate, and 0.1 μmol SAM. Malonyl-CoA or glutaryl-CoA were added at 0.2 μmol, whereas 50 μg pimeloyl-ACP or another acyl-ACP substrate was added. The reactions were incubated at 37 °C for 4 h and quenched by immersion in boiling water for 10 min. DTB/biotin production was assayed by addition of 10 μl reaction mixture onto the paper disks. KAPA synthesis was assayed in a similar manner except that only purified BioF proteins from *E. coli* and *B. subtilis* were used. Note that DTB bioassay is five to sixfold less sensitive than bioassay of biotin^27,52^.

### LC-MS/MS analysis of the modified active site peptide of BioZ C115S

These analyses were performed by the Protein Sciences Facility of the Roy J. Carver Biotechnology Center, University of Illinois. The BioZ C115S mutant protein was digested with Glu-C and trypsin in 50 mM ammonium acetate. Liquid chromatography-mass spectrometry analysis was perform using a Dionex Ultimate 3000 RSLC nanoflow UPLC system (Thermo Scientific) coupled to a Fusion Tribrid Orbitrap mass spectrometer (Thermo Scientific). An Acclaim PepMap RSLC column (75 µm × 15 cm) was used for the separation using a 60 min gradient (1–60%) of acetonitrile in 0.1% formic acid. The spectra were obtained using the Universal method utilizing data-dependent acquisition and maximum parallelizable throughput. The collected spectra were processed using Mascot Distiller and analyzed using MASCOT run against a customized database containing the sequence of the protein of interest.

### Mass spectra of acyl-ACP species

Strep-tagged apo, holo, acetyl, malonyl, glutaryl, and pimeloyl-ACP standards were synthesized and purified as were the untagged ACP species described above, followed by a Strep-Tacin XT column purification based on the protocol of the manufacturer before use in BioZ reactions. ACP proteins from BioZ reactions were purified from the reaction with a column containing Strep-Tactin resin and after elution buffer exchanged into 50 mM ammonium bicarbonate using 3000 Da molecular weight cutoff spin columns. A Q Exactive HF-X Hybrid Quadrupole-Orbitrap instrument was used for intact analysis of ACP species by the Protein Sciences Facility of the Roy J. Carver Biotechnology Center, University of Illinois. The instrument was operated at 120k resolution in the high mass range mode with a scan range of 700–3000 m/z. Standard and samples were separated with a C5 column from 20% to 60% acetonitrile in 0.1% formic acid at 150 μL/min for 15 min. The data were deconvoluted using the Xtract algorithm and averaged all the spectra across the retention time and further analyzed by BioPharma Finder 2.0 (Thermo).

### HPLC of acyl-CoA species

Acyl-CoA species from the reaction were first separated from high molecular weight components using 3000 Da cutoff spin columns, then equilibrated with two volumes of 150 mM ammonium acetate/acetate buffer pH 4.5 containing 4% acetonitrile before injection into the high-performance liquid chromatography (HPLC). Acyl-CoA standards were purchased from Sigma-Aldrich then made into 10 mM stock in water. A Beckman System Gold HPLC system with an Agilent Pursuit XRs Ultra C18 column were used to separate the acyl-CoA species using a 150 mM ammonium acetate/acetate buffer pH 4.5 and a 4–24% acetonitrile non-linear gradient at 1 ml/min for 45 min. The gradients of acetonitrile were: 0–5 min 4%, 5–10 min linear increases from 4% to 16%, 10–35 min linear increases from 16% to 24%, 35–38 min remain at 24%, 38–40 min decrease from 24% to 4%, 40–45 min remain at 4%. In this method, a series of mixture of CoA and acyl-CoA standards were run to calibrate the separations. The approximate retention times were: CoA, 13.5 min; acetyl-CoA, 15.5 min; malonyl-CoA, 8 min; succinyl-CoA, 15 min; glutaryl-CoA, 16 min, and pimeloyl-CoA, 18.5 min.

### Biotin synthesis dependent on supplementation of resting *A. tumefaciens* cell cultures

All *A. tumefaciens* strains were grown at 28 °C. To make resting cell suspensions, the biotin regulation deletion strain *A. tumefaciens* NTL4 *∆bioR*::Km (BioR is a weak repressor of biotin synthesis^30^) were grown in 300 ml of AT minimal medium with 2 nM biotin at 28 °C to OD_600_ 0.8. The cells were harvested and washed in AT salts four times to remove biotin, then cell growth was arrested by sub-culturing in 600 ml AT minimal media lacking a carbon source for 6 h. Avidin (0.1 units/ml) was added to the medium to prevent cross-feeding and neutralize any biotin or DTB contamination. Cells were collected by centrifugation at 3700 × *g* for 10 min and washed four times with AT salts to remove avidin. The cell pellets were suspended in 600 ml AT minimal medium lacking a carbon source. Ten ml aliquots were added to flasks containing different concentrations of either an α,ω-dicarboxylic acid, 2-oxoadipate, or l-lysine. The cultures were incubated at 28 °C with shaking for 12–16 h. The cells were removed by centrifugation and the clear culture from each feeding were used for bioassay on plates of *E. coli* strain ER90 as above.

### Reporting summary

Further information on research design is available in the Nature Research Reporting Summary linked to this article.

## Supplementary information

Supplementary Information

Reporting Summary

## Data Availability

The data sets generated during and/or analyzed during the current study are available from the corresponding author on reasonable request. Source data are provided with this paper.

## Source data

Source Data
