# Supplementary material for: α-proteobacteria synthesize biotin precursor pimeloyl-ACP using BioZ 3-ketoacyl-ACP synthase and lysine catabolism
Source: Nat Commun. 2020 Nov 5;11:5598. doi: 10.1038/s41467-020-19251-5 (PMC7645780; doi:10.1038/s41467-020-19251-5)
Supplement: Supplementary file 1 — Supplementary Information [file 41467_2020_19251_MOESM1_ESM.pdf]

Supplementary Information to:

α-Proteobacteria Synthesize Biotin Precursor Pimeloyl-ACP Using the BioZ 3-Ketoacyl-ACP Synthase and Lysine Catabolism.

Yuanyuan Hu and John E. Cronan

Supplementary Table 1. Oligonucleotide primers

Primer (5'-3')	Sequence
A.t bioZ-Nco	ATTCCATGGTGATGCAGACACGTTCTTCC
A.t bioZ-Pst	TATCTGCAGTTAGACGCGATAAACCAC
A.t bioZ-Nde	TAACATATGCAGACACGTTCTTCCC
A.t bioZ-Xho	TATCTCGAGTTAGACGCGATAAACCAC
A.t bioZ C115S F	ATCGATCTTGCCGGGGCCTCCTCCGGGTTTCTTTATG
A.t bioZ C115S R	CATAAAGAAACCCGGAAGGGGCCCCGGCAAGATCG
B.a bioZ-NdeI	ATACATATGACGGTCTGTTCCAG
B.a bioZ-HindIII	TATAAGCTTTCATACCTGCATCAGCAC
B.a bioZ-NcoI	ATTCCATGGTGATGACGGTCTGTTCCAG
M.j bioZ-Pci	ATTACATGTTGATGAGCAAGTCGTCGCGCATTC
M.j bioZ-Hind	TTAAAGCTTCTAAATTCCAACGACGAGCGCAC
M.j bioZ-NdeI	TAACATATGAGCAAGTCGTCGCGC
R.m bioZ- NdeI	ATTCATATGTTGCCTGAACAGTCC
R.m bioZ-HindIII	ATAAAGCTTACCATTGGATCAGCAC
R.m bioZ-NcoI	ATTCCATGGTGATGTTGCCTGAACAGTCC
M.h bioZ-NcoI	ATTCCATGGTGAGCACGGCAGGC
M.h bioZ-Hinstop	ATAAAGCTTCTACCAGCGCAGCACCG
M.h bioZ-NdeI	ATACATATGGTGAGCACGGCAGGC
S.f bioZ-Nco	ATTCCATGGTGAGTACCATTCGCTCCTCC
S.f bioZ-Hin	ATTAAGCTTCTAAAGTCCGACGACCACC
A.t fabH-PciI	TAAACATGTATGATCCGCTCTATAGTCCG
A.t fabH-Hin	TATAAGCTTTTACCAGCGCAGCAGC
B.a fabH-PciI	ATTACATGTTGATGATAAGATCTGTCGTACGGGGTA
B.a fabH-Pst	TATCTGCAGTTACCAGCGCACAAGAACCG

R.m fabH-Nco	ATTCCATGGTGATGCCCTACGCAGCCATTACTTC
R.m fabH-Hin	TATAAGCTTTCAGGGGTTTGTGGTGGACG
M.h fabH-Nco	AGTCCATGGCCGCAGGGCTC
M.h fabH-Hin	ATAAAGCTTACGCTTCACCTCCGTACCAG
S.f fabH-Nde	ATACATATGATCCGTTCTGTCGTTCG
S.f fabH-Hind	TATAAGCTTTTACCAGCGGATGAGAAC
E.c fabH-Pcil	AATACATGTATACGAAGATTATTGGTACTGGC
E.c fabH-Hin	AATAAGCTTAGAAACGAACCAGCG
A.t bioZKO Eco	AATGAATTCTTATTCCGATAGCGGTTCAACGGCG
A.t bioZKO upR	TGTCAGGACTTTCGCAATGGTC TGG
A.t bioZKO downF	AAGTCCTGACATAATAAGCCCACCCTTAGCAATTGC
A.t bioZKO Pst	TTACTGCAGCCTTCAACCGGACAAGGCAGC
A.t fabH insert Eco	ATCGAATTCCGACATGCAGGCCGTCTG
A.t fabH insert Pst	TATCTGCAGTCCCGGCCTTCCATGC
A.t caiBKO Pst	ATACTGCAGATGATGAATGAGCCGACCGATAAC
A.t caiBKO upR	TTTATCTCATGGACTTATTCCGTTTC
A.t caiBKO dwnF	ATGAAGGCAGGAATGAAAAGAACTG
A.t caiBKO Hin	TATAAGCTTCACATTGGCCTCGATCCAGTC

The restriction sides used for insertion into the various vector sites (generally NcoI or NdeI for the upstream sequences and HindIII, PstI or SalI for the downstream) are underlined.

Supplementary Table 2. Bacterial Strains

**Strains**	**Strain description**	**Reference**
	E. coli K-12 strains (all are MG1655 derivatives, except ER90, R878 & NRD25)	
MG1655	wild type strain	CGSC
R878	*bioC23*	CGSC
STL23	*ΔbioC::FRT*	[1]
STL24	*ΔbioH::FRT*	[1]
STL25	*ΔbioC::FRT ΔbioH::FRT*	[1]
YUAN230	pCY125/*ΔbioC::FRT ΔbioH::FRT* pBR329 carrying the *bioABFCD* operon with in-frame *bioC* deletion.	[1]
YUAN090	pBAD322C/*ΔbioC::FRT*	This study
YUAN091	pBAD322C/*ΔbioH::FRT*	This study
YUAN092	pBAD322C/ *ΔbioC::FRT ΔbioH::FRT*	This study
YUAN139	pBAD322C-*AtbioZ*/*ΔbioC::FRT*	This study
YUAN140	pBAD322C-*AtbioZ*/*ΔbioH::FRT*	This study
YUAN141	pBAD322C-*AtbioZ ΔbioC::FRT ΔbioH::FRT*	This study
YUAN097	pBAD322C-*BabioZ*/*ΔbioC::FRT*	This study
YUAN098	pBAD322C-*BabioZ*/*ΔbioH::FRT*	This study
YUAN099	pBAD322C-*BabioZ*/*ΔbioC::FRT ΔbioH::FRT*	This study
YUAN148	pBAD322A-*MjbioZ*/*ΔbioC::FRT*	This study
YUAN149	pBAD322A- *MjbioZ* /*ΔbioH::FRT*	This study
YUAN150	pBAD322A- *MjbioZ*/*ΔbioC::FRT ΔbioH::FRT*	This study
YUAN125	pBAD322C-*MhbioZ*/*ΔbioC*::FRT	This study
YUAN126	pBAD322C- *MhbioZ* /*ΔbioH*::FRT	This study
YUAN127	pBAD322C- *MhbioZ* /*ΔbioC::FRT ΔbioH::FRT*	This study
YUAN111	pBAD322C-*RmbioZ*/*ΔbioC::FRT*	This study
YUAN112	pBAD322C- *RmbioZ* /*ΔbioH::FRT*	This study
YUAN113	pBAD322C- *RmbioZ* /*ΔbioC::FRT ΔbioH::FRT*	This study
YUAN214	pBAD322C-SfbioZ/ *ΔbioC::FRT*	This study
YUAN215	pBAD322C-SfbioZ/ *ΔbioH::FRT*	This study
YUAN216	pBAD322C-SfbioZ/ *ΔbioC::FRT ΔbioH::FRT*	This study
YUAN301	pBAD322C-*AtfabH*/*ΔbioC::FRT*	This study

YUAN302	pBAD322C- *AtfabH* /*ΔbioH*::*FRT*	This study
YUAN303	pBAD322C- *AtfabH* /*ΔbioC::FRT* ΔbioH::FRT	This study
YUAN104	pBAD322C-*BafabH*/*ΔbioC::FRT*	This study
YUAN105	pBAD322C- *BafabH*/*ΔbioH::FRT*	This study
YUAN106	pBAD322C- *BafabH*/*ΔbioC::FRT ΔbioH::FRT*	This study
YUAN132	pBAD322C-*MhfabH*/*ΔbioC::FRT*	This study
YUAN133	pBAD322C- *MhfabH* /*ΔbioH::FRT*	This study
YUAN134	pBAD322C- *MhfabH* /*ΔbioC::FRT ΔbioH::FRT*	This study
YUAN118	pBAD322C-*RmfabH*/*ΔbioC::FRT*	This study
YUAN119	pBAD322C- *RmfabH* /*ΔbioH::FRT*	This study
YUAN120	pBAD322C- *RmfabH* /*ΔbioC::FRT ΔbioH::FRT*	This study
BL21 (DE3)	F- *ompT hsdSB gal dcm* (DE3)	Invitrogen
YUAN235	pET28b-*EcfabH*/ BL21 (DE3)	Lab stock
YUAN220	pET16b-*EcfabG*/ BL21 (DE3)	Lab stock
YUAN222	pET16b-*EcfabA*/ BL21 (DE3)	Lab stock
YUAN221	pET16b-*EcfabI*/ BL21 (DE3)	Lab stock
STL9	pET28b-*EcbioA*/ BL21 (DE3)	[2]
STL12	pET28b-*EcbioD*/ BL21 (DE3)	[2]
STL14	pET28b-*EcbioH*/ BL21 (DE3)	[2]
STL13	pET28b-*EcbioF*/ BL21 (DE3)	[2]
YFJ239	pYFJ84 (pET16-*aasS*)/ BL21 (DE3) p*LysS*	[3]
YUAN236	pET28b-*sfp*/ BL21 (DE3)	This study
YUAN225	pJT94 (pDHC30-*acpH*) /DK574	This study
YUAN226	pJT93 (pDHC30-*acpS*) / DK574	This study
YUAN170	pET28b-*AtbioZ*/ BL21 (DE3)	This study
YUAN174	pET28b-*MlbioZ*/ BL21 (DE3) Tuner	This study
YUAN395	pET28b-*SfbioZ*/ BL21 (DE3) Tuner	This study
YUAN299	pET28b-*AtfabH*/ BL21 (DE3) Tuner	This study
YUAN435	pET28b-*SffabH*/ BL21 (DE3) Tuner	This study
YUAN393	pET30a-AtcaiB/ BL21 (DE3) Tuner	This study
YUAN515	pET28b-*acpP*-Strep-tag, pJT94/ BL21(DE3)	This study
BL21 Rosetta pLysS	F^- *ompT* *hsdS*$_B$(r_B^- m_B^-) *gal dcm* (DE3) pLysSRARE (Cam^R)	Novagen

YUAN161	pET30a/ BL21 Rosetta pLysS	This study
YUAN168	pET30a-*BabioZ*/ BL21 Rosetta pLysS	This study
YUAN182	pET30a-*MhbioZ*/ BL21 Rosetta pLysS	This study
YUAN178	pET30a-*RmbioZ*/ BL21 Rosetta pLysS	This study
YUAN190	pET30a-*MhfabH*/ BL21 Rosetta pLysS	This study
YUAN188	pET30a-*RmfabH*/ BL21 Rosetta pLysS	This study
ER90	MC1061 *bioF* inactivated by Cm cassette insertion polar inactivation of *bioC* and *bioD.*	[5]
NRD25	MC1061 Δ*(bioABFCD)*	Lab stock
STL108	MG1655 *ΔbioF bioH*::Kan	[2]
	A. tumefaciens C58 strains (except FYJ212)	
C58	Wild type strain	S. Farrand
YUAN371	*ΔbioZ*	This study
YUAN399	*bioZC115S*	This study
YUAN373	*fabH::pK19mobsacB*	This study
YUAN405	*ΔcaiB*	This study
YUAN409	pSRKGm/*ΔbioZ*	This study
YUAN419	*fabH::*pK19*mobsacB*	This study
YUAN411	pSRKGm-*AtbioZ*/*ΔbioZ*	This study
YUAN413	pSRKGm-*BabioZ*/*ΔbioZ*	This study
YUAN415	pSRKGm-*SfbioZ*/*ΔbioZ*	This study
FYJ212	*A. tumefaciens NTL4 ΔbioR*	[6]

FRT is the flip recombination site. CGSC is the Coli Genetic Stock Center (Yale).

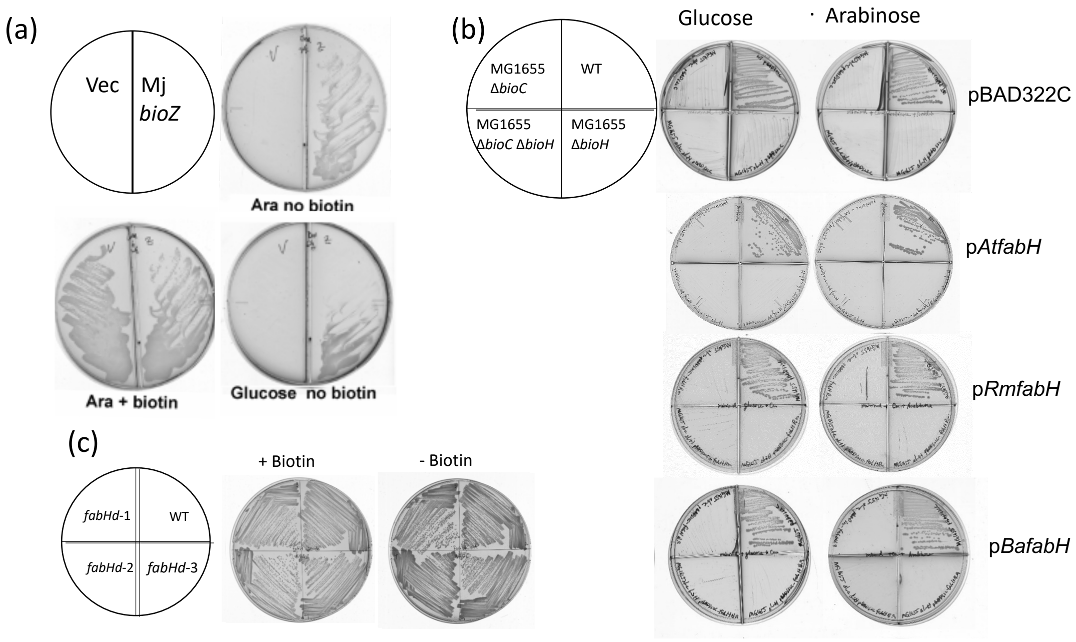

Supplementary Fig 1. **(a)** Complementation of *bioC23* strain R878 with *M. japonicum bioZ***.** The gene was expressed from an arabinose promoter (Vec denotes the empty vector) **(b)** Failure of *fabH* plasmids to complement *E.coli* MG1655 *ΔbioC,* Δ*bioC ΔbioH or bioH* mutant strains. **(c)** *A. tumefaciens fabH* disruption strains do require biotin for growth Three colonies were streaked. WT denotes the wild type strain.

Imidazole concentrations (in mM): E1, 62.5; E2, 125; E3, 250 and E4, 500.

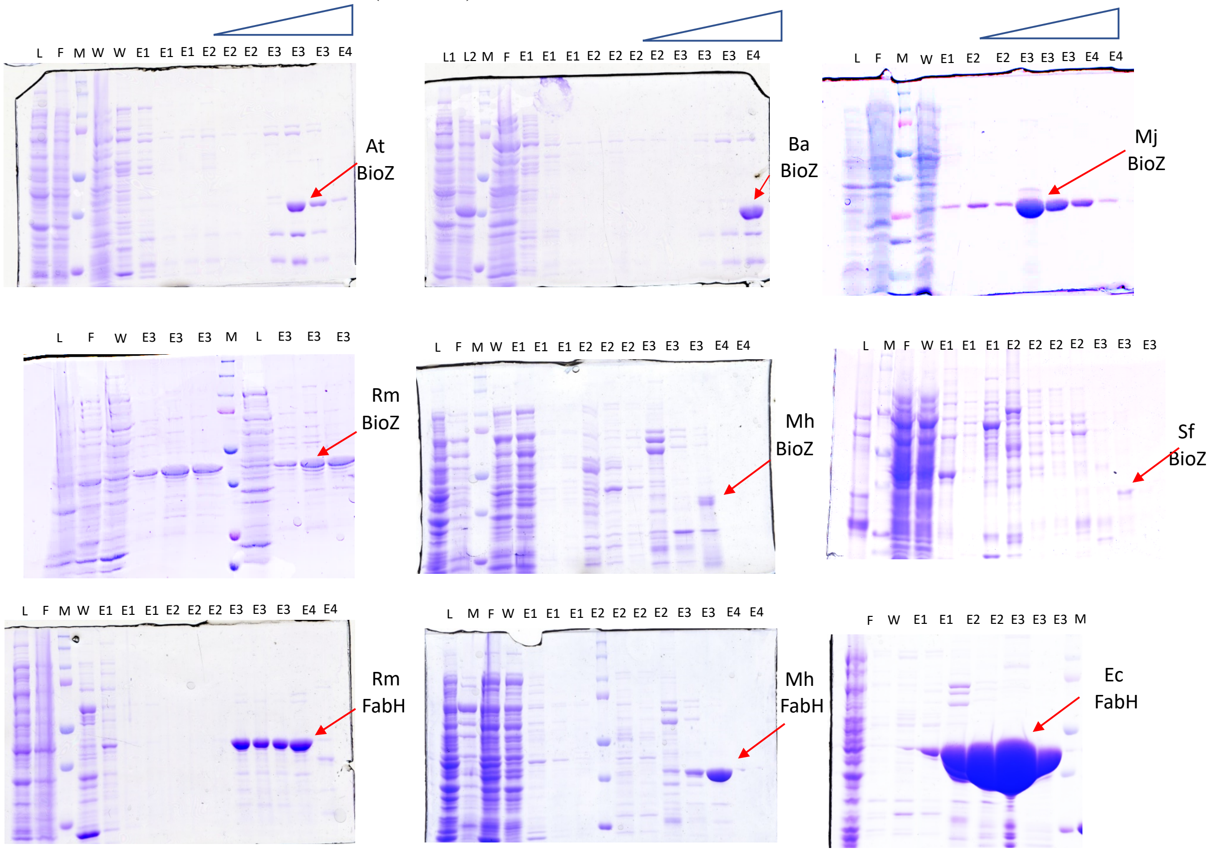

Supplementary Fig. 2. Purification of BioZ proteins and *E. coli* (Ec) FabH. Sodium dodecyl sulfate gels of representative fractions eluted from the nickel chelate columns are shown in the right-hand lanes of the gels whereas the left-hand lanes show uninduced and induced extracts, a molecular weight standard and early eluate fractions, respectively. The red arrows denote the final purified protein. L-lysate, F-flowthrough, M-protein ladder, W-wash, E1-elution at 8% buffer B, contains final concentration of 62.5 mM imidazole, E2-elution at 16% buffer B, contains final concentration of 125 mM imidazole, E3-elution at 50% buffer B, contains final concentration of 250 mM imidazole, E4-elution at 100% buffer B, contains final concentration of 500 mM imidazole. The imidazole gradients are depicted by the triangles at the top of the figure. The protein bands below the At and Ba BioZ bands are heat shock chaperones that copurify with BioZ. Strain abbreviations as in Fig. 2.

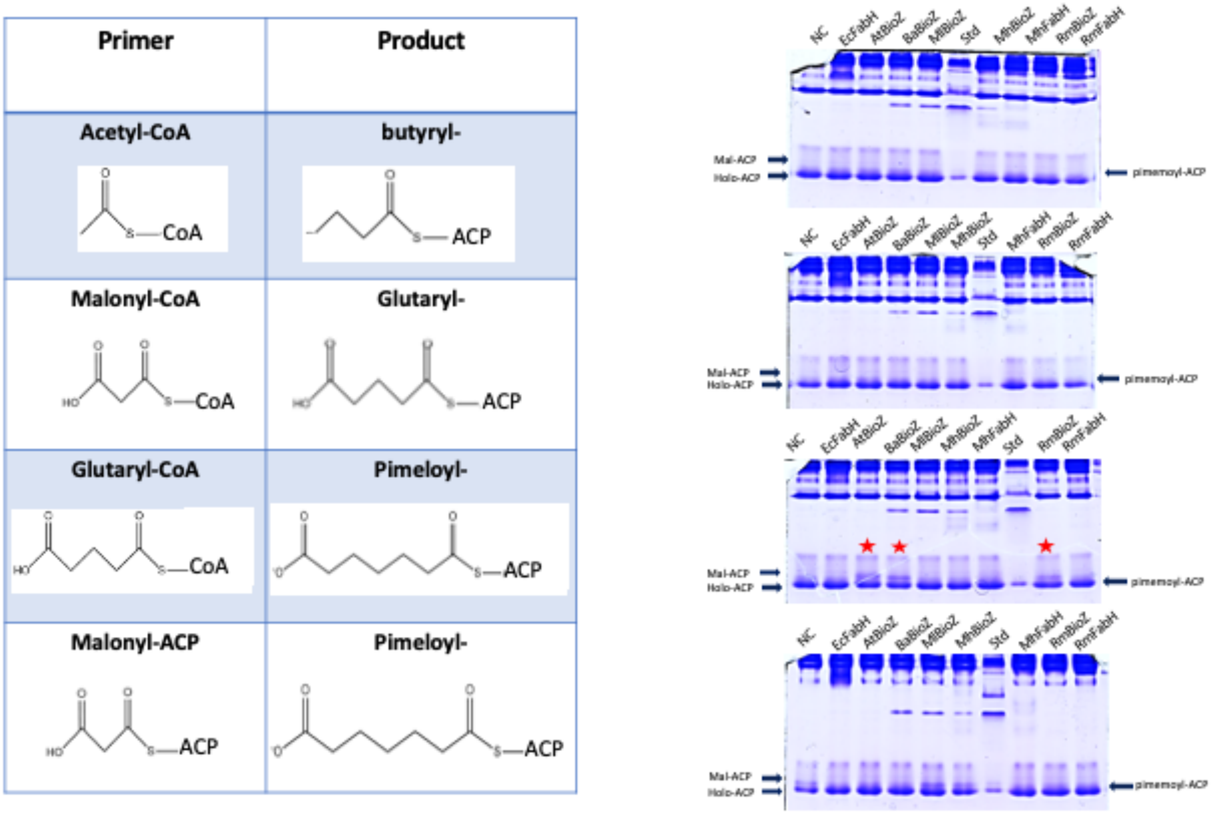

Supplementary Fig. 3. Gel-shift analyses of BioZ *in vitro* reactions with *E. coli* fatty acid biosynthesis proteins. Testing of various primer substrates in BioZ-catalyzed condensation with malonyl-ACP. BioZ condensed malonyl-ACP with glutaryl-CoA, but not with acetyl-CoA, malonyl-CoA or malonyl-ACP. The red asterisk denote reactions in which pimeloyl-ACP was produced. Each experiment was repeated >3 times and the gels given are representative.

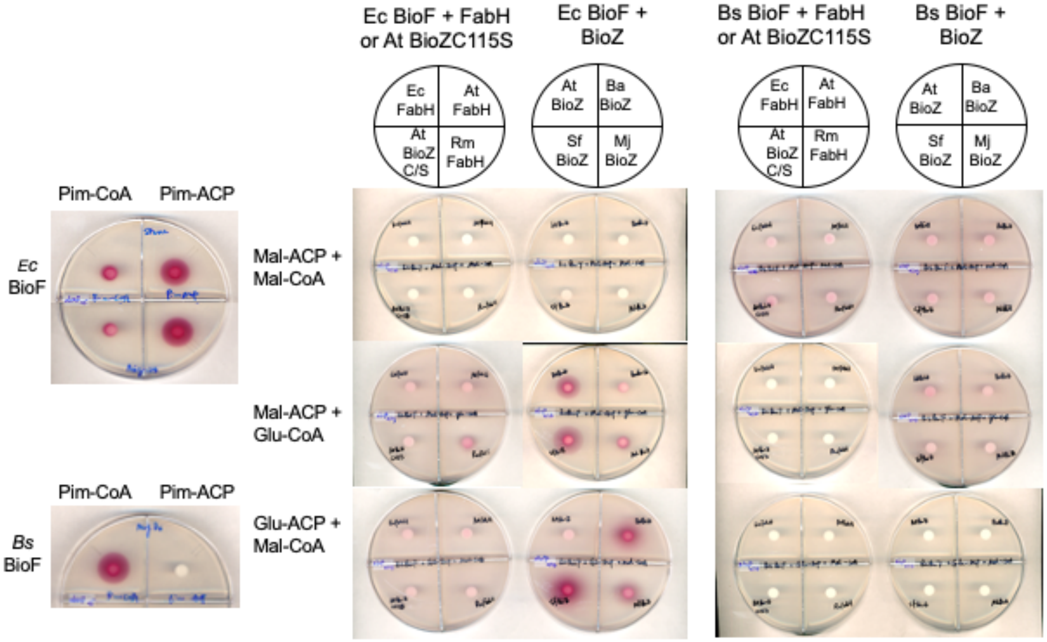

Supplementary Fig. 4 The BioZ product formed *in vitro* is pimeloyl-ACP

KAPA (7-oxo-8-aminopelargonic acid), the first intermediate in synthesis of th e heterocyclic rings of biotin, is synthesized by a pyridoxal 5'-phosphate-dependent condensation of pimeloyl-CoA or pimeloyl-ACP with L-alanine accompanied by decarboxylation of L-alanine and release of the thiol moiety [7]. Previous studies have shown that *B. subtilis* BioF specifically accepts only pimeloyl-CoA as acyl donor whereas pimeloyl-ACP is inactive both *in vivo* and *in vitro* [8]. In contrast *E. coli* BioF accepts either of the pimeloyl-thioesters [8]. To determine whether the pimeloyl moiety formed by *in vitro* BioZ reactions is attached to CoA or to ACP, either *B. subtilis* BioF or *E. coli* BioF was used for *in vitro* pathway reconstitutions. KAPA synthesis was detected by bioassay using an *E. coli* Δ*bioF* strain. KAPA formation was detected only in the reactions that contained *E. coli* BioF, indicating that the *in vitro* BioZ reaction product is pimeloyl-ACP.

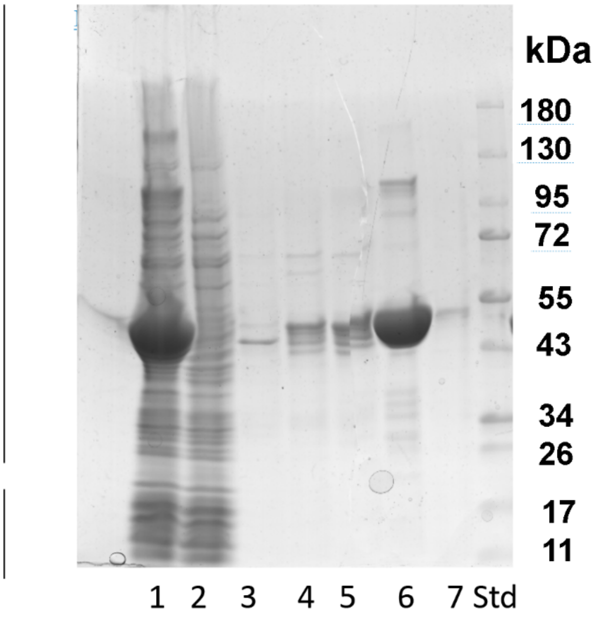

Supplementary Fig. 5 ***A. tumefaciens*** **CaiB purification**

Sodium dodecyl sulfate gels of steps in the standard purification protocol (Material and Methods) of *A. tumefaciens* CaiB. Lane 1, Induced crude extract; lane 2 column flow through. Lanes 3-6 progressive elution of the column with increasing imidazole concentrations as in Supplementary Figure 2. The right-hand lane is the molecular weight standard. The purification was performed twice with excellent yields.

References.

1. Lin S, Hanson RE, Cronan JE. Biotin synthesis begins by hijacking the fatty acid synthetic pathway. *Nat Chem Biol* **6**, 682-688 (2010).

2. Lin S. Biotin synthesis in *Escherichia coli* Ph.D. Thesis, Uiversity of Illinois, Urbana-Champaign (2018)
.
3. Jiang Y, Morgan-Kiss RM, Campbell JW, Chan CH, Cronan JE. Expression of *Vibrio harvey*i acyl-ACP synthetase allows efficient entry of exogenous fatty acids into the *Escherichia coli* fatty acid and lipid A synthetic pathways. *Biochemistry* **49**, 718-726 (2010).

4. Cronan JE, Thomas J. Bacterial fatty acid synthesis and its relationships with polyketide synthetic pathways. *Methods Enzymol* **459**, 395-433 (2009).

5. Choi-Rhee E, Cronan JE. A nucleosidase required for in vivo function of the S-adenosyl-L-methionine radical enzyme, biotin synthase. *Chem Biol* **12**, 589-593 (2005).

6. Feng Y, Zhang H, Cronan JE. Profligate biotin synthesis in alpha-proteobacteria - a developing or degenerating regulatory system? *Mol Microbiol* **88**, 77-92 (2013).

7. Mann S, Ploux O. Pyridoxal-5'-phosphate-dependent enzymes involved in biotin biosynthesis: structure, reaction mechanism and inhibition. *Biochim Biophys Acta* **1814**, 1459-1466 (2011).

8. Manandhar M, Cronan JE. A canonical biotin synthesis enzyme, 8-amino-7-oxononanoate synthase (BioF), utilizes different acyl chain donors in *Bacillus subtilis* and *Escherichia coli*. *Appl Environ Microbiol* **84**, (2018).
